# Machine Learning for Modeling the Singular Multi-Pantograph Equations

**DOI:** 10.3390/e22091041

**Published:** 2020-09-18

**Authors:** Amirhosein Mosavi, Manouchehr Shokri, Zulkefli Mansor, Sultan Noman Qasem, Shahab S. Band, Ardashir Mohammadzadeh

**Affiliations:** 1 Environmental Quality, Atmospheric Science and Climate Change Research Group, Ton Duc Thang University, Ho Chi Minh City, Vietnam; amirhosein.mosavi@tdtu.edu.vn; 2 Faculty of Environment and Labour Safety, Ton Duc Thang University, Ho Chi Minh City, Vietnam; 3 Faculty of Civil Engineering, Institute of Structural Mechanics (ISM), Bauhaus-Universität Weimar, 99423 Weimar, Germany; Manouchehr.shokri@uni-weimar.de; 4 Fakulti Teknologi dan Sains Maklumat, Universiti Kebangsan Malaysia, Bangi 43600, Selangor, Malaysia; kefflee@ukm.edu.my; 5 Computer Science Department, College of Computer and Information Sciences, Al Imam Mohammad Ibn Saud Islamic University (IMSIU), Riyadh 11432, Saudi Arabia; SNMohammed@imamu.edu.sa; 6 Computer Science Department, Faculty of Applied Science, Taiz University, Taiz 6803, Yemen; 7 Future Technology Research Center, National Yunlin University of Science and Technology, 123 University Road, Section 3, Douliou, Yunlin 64002, Taiwan; 8 Institute of Research and Development, Duy Tan University, Da Nang 550000, Vietnam; 9 Electrical Engineering Department, University of Bonab, Bonab 5551785176, Iran; a.mzadeh@ubonab.ac.ir

**Keywords:** fuzzy systems, square root cubature kalman filter, singular multi-pantograph differential equations, statistical analysis, Lyapunov function

## Abstract

In this study, a new approach to basis of intelligent systems and machine learning algorithms is introduced for solving singular multi-pantograph differential equations (SMDEs). For the first time, a type-2 fuzzy logic based approach is formulated to find an approximated solution. The rules of the suggested type-2 fuzzy logic system (T2-FLS) are optimized by the square root cubature Kalman filter (SCKF) such that the proposed fineness function to be minimized. Furthermore, the stability and boundedness of the estimation error is proved by novel approach on basis of Lyapunov theorem. The accuracy and robustness of the suggested algorithm is verified by several statistical examinations. It is shown that the suggested method results in an accurate solution with rapid convergence and a lower computational cost.

## 1. Introduction

The application of multi-pantograph differential equations (MDEs) is expanding into various branches of science such as modeling of cell-growth [1], electrodynamics [2], number theory [3], electrodynamics, astrophysics [4], atomic physics [5], among many others.

Recently, due to the importance of MDEs, the solving of these equations have been frequently considered in the literature and many numerical and analytical methods have been presented [6]. For example, in References [7,8,9], a homotopy approach and power series are developed for solving linear MDEs and coinciding of the estimated solution with the exact solution is investigated. In Reference [10], the spectral tau method is studied and the convergence of the presented approach is investigated by $L^{2}$ norm. In Reference [11], by obtaining the fractional integral of Taylor wavelets in the sense of Riemann-Liouville definition, an estimated solution is presented for fractional MDEs. In Reference [12], by the Bessel and block-pulse functions, a numerical solution is suggested and it is shown that the increasing of Bessel functions improves the accuracy. In Reference [13], on the basis of topological degree theorem, an analytical method is developed. In Reference [14], by the use of residual power series, an analytical solution is obtained and the efficiency of using residual power series is compared with Chebyshev and Boubaker polynomials. In Reference [15], Adomian decomposition approach is used to construct a solution algorithm for MDEs with fractional-order in the sense of Caputo definition. In Reference [16], by the use of Riemann Liouville fractional derivative and integral definitions, some operational matrices are constructed and then on basis of Jacobi polynomials, an analytical solution is presented. In Reference [17], the collocation method by the use of Boubaker polynomials is developed to convert the problem into a nonlinear system, and then by solving the reduced nonlinear system, an approximated solution is presented. In Reference [18], the spectral tau approach using Jacobi polynomials is improved to solve MDEs. The shifted Gegenbauer-Gauss collocatio technique is introduced in Reference [19], for functional-differential equations. The exponential Jacobi spectral and Jacobi collocation approaches are developed in References [20,21].

Recently, fuzzy logic systems (FLSs) and machine learning algorithms are widely applied on engineering problems [22,23,24,25,26,27,28]. However, to the best knowledge of the authors, the solution of singular MDEs by FLSs and machine learning algorithms has not been studied in the literature. However, quite rarely, some neural methods have been presented for conventional MDEs. For example, in Reference [29], a neural network (NN) is learned by genetic algorithm to find a solution for a pantograph system. In Reference [30], a simple NN is used to find an approximated solution for ordinary differential equations and its accuracy is compared with the analytical solution. In Reference [31], by the method of Lagaris et al, a neural approach is developed for solving MDEs and its efficiency is proved. Now days, the computation techniques in software engineering are used in various branch of scientific problem such as multimedia systems [32,33], security systems [34], forecasting problems [35], stock market prediction [36], control systems [37], internet of things [38], and so on. However these effective techniques quite rarely are applied on MDEs.

Considering the above motivation, in this paper a new approach using T2-FLSs is presented for the solving of singular MDEs. Unlike the aforementioned NN-based methods [29,30,31], the optimization is done by a low computation cost and stable algorithm. The proposed learning algorithm is on the basis of stable SCKF. For the first time, a new approach on the basis of the Lyapunov theorem is suggested to analyze the convergence and closed-loop stability. By several statistical examinations, such as root mean square error (RMSE), inequality coefficient of Theil index (TIC), variance (VAR), fitness (FIT), interquartile range (IR), median (Med), minimum (Min) and mean of absolute error, the accuracy of the suggested method is shown. The main contributions are:

- A new numerical method is proposed for solving singular MDEs.
- For the first time, a type-2 fuzzy logic based approach is formulated to find an approximated solution.
- A new approach on the basis of the Lyapunov theorem is introduced for convergence and stability analysis.
- Square root cubature Kalman filter is developed for the optimization of the suggested solver.
- Several statistical examinations are presented to demonstrate the accuracy and stability.

The paper organization is as follows. The problem is formulated in Section 2. The suggested T2-FLS is illustrated in Section 3. The learning algorithm is presented in Section 4. The stability is investigated in Section 5. The evaluation indexes are described in Section 6. The simulation results are provided in Section 7, and finally the main outcomes are summarized in Section 8.

## 2. Problem Formulation

**Figure 1:**
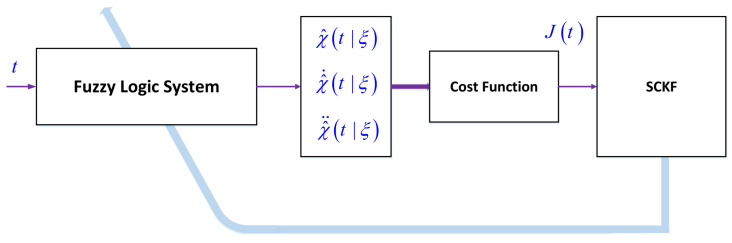
Block diagram of the proposed solver.

The suggested solver is designed on the basis of fuzzy systems and SCKF. The general diagram of the suggested solution approach is shown in Figure 1. The problem is described as:

(1) $$\ddot{\chi }\left(t\right)+\sum_{k=1}^{n}\dot{\chi }\left(r_{k}t\right)/P_{k}\left(t\right)+\chi \left(t\right)/G_{k}=F\left(t\right),$$

where the initial conditions are $\chi \left(0\right)=a_{1}$ and $\dot{\chi }\left(0\right)=a_{2}$. $F_{k}\left(t\right)$ and $G\left(t\right)$ are nonlinear functions. If there is a singularity in $F_{k}\left(t\right)$ and $G\left(t\right)$, then both sides of (1) are multiplied by $F_{k}\left(t\right)$ and $G\left(t\right)$. The parameters of T2-FLS should be learned such that the estimated solution $\hat{\chi }\left(t\right)$ is to be converged to the exact solution $\chi \left(t\right)$. Then the estimated $\hat{\chi }\left(t\right)$ satisfies:

(2) $$\ddot{\hat{\chi }}\left(t\right)+\sum_{k=1}^{n}\dot{\hat{\chi }}\left(\gamma_{k}t\right)/P_{k}\left(t\right)+\hat{\chi }\left(t\right)/G_{k}=F\left(t\right).$$

The cost function is defined as follows:

(3) $$J=\frac{1}{N}\sum_{i=1}^{N}{\left({\ddot{\hat{\chi }}}_{i}\left(t\right)+\sum_{k=1}^{n}{\dot{\hat{\chi }}}_{i}\left(\gamma_{k}t\right)/P_{k,i}\left(t\right)+{\hat{\chi }}_{i}\left(t\right)/G_{i}-F\left(t\right)\right)}^{2}+\frac{1}{2}\left({\hat{\chi }}_{0}^{2}+{\dot{\hat{\chi }}}_{0}^{2}\right),$$

where i=1,…,N and *N* is the number of samples.

## 3. T2-FLS Structure

**Figure 2:**
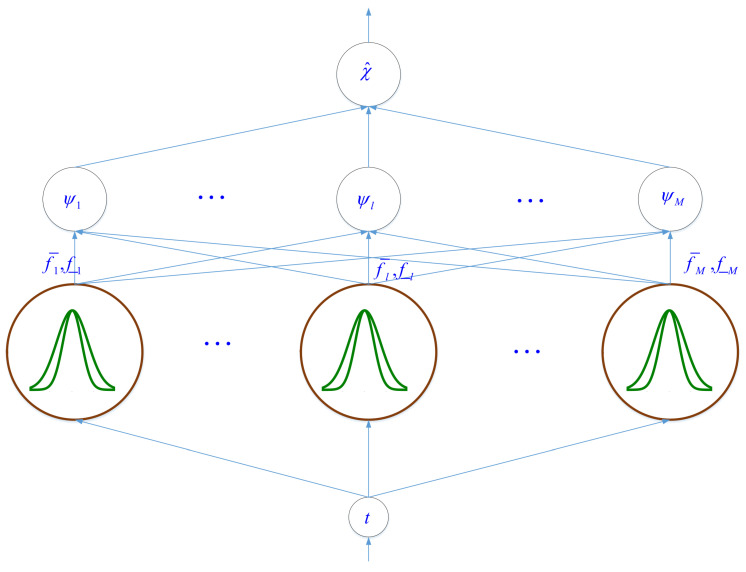
The structure of suggested T2-FLS.

The structure of T2-FLS $\hat{\chi }\left(t\right)$ is shown in Figure 2. The details are given as follows:

(1) Get the input *t*.
(2) The input *t* is mapped into time range $\left[0,1\right]$.
(3) The $\left[0,1\right]$ is divided into *M* section and for each section a Gaussian membership function (MF) with mean $m_{l},\;l=1,\ldots ,M$ and variance $\nu_{l}$ is considered.
(4) The upper and lower firing rules are computed as:
  (4) $${\bar{f}}_{l}\left(t\right)=\exp\left(-\frac{{\left(t-m_{l}\right)}^{2}}{{\bar{\nu }}_{l}^{2}}\right),l=1,\ldots ,M,$$
  (5) $${\underline{f}}_{l}\left(t\right)=\exp\left(-\frac{{\left(t-m_{l}\right)}^{2}}{{\underline{\nu }}_{l}^{2}}\right),l=1,\ldots ,M.$$
(5) The normalized rule firings (type-reduction by the Nie-Tan approach [39]) are obtained as:
  (6) $$\psi_{l}\left(t\right)=\frac{{\bar{f}}_{l}\left(t\right)+{\underline{f}}_{l}\left(t\right)}{\sum_{l=1}^{M}{\bar{f}}_{l}\left(t\right)+{\underline{f}}_{l}\left(t\right)}.$$
(6) The output is obtained as:
  (7) $$\hat{\chi }\left(t,\theta ,m,\nu \right)=\sum_{l=1}^{M}\theta_{l}\psi_{l}\left(t,m,\nu \right),$$
  where *M* is number of rules, θ is the vector of rule parameters. From (7), $\dot{\hat{\chi }}$ is computed as:
  (8) $$\dot{\hat{\chi }}\left(t,\theta ,m,\nu \right)=\sum_{l=1}^{M}\theta_{l}\frac{{\dot{f}}_{l}\left(t\right)\sum_{l=1}^{M}f_{l}\left(t\right)-f_{l}\left(t\right)\sum_{l=1}^{M}{\dot{f}}_{l}\left(t\right)}{{\left(\sum_{l=1}^{M}f_{l}\left(t\right)\right)}^{2}}.$$
  From (8), ${\dot{f}}_{l}\left(t\right)$ is:
  (9) $${\dot{f}}_{l}\left(t\right)=-\frac{2\left(t-m_{l}\right)}{\nu_{l}^{2}}\exp\left(-\frac{{\left(t-m_{l}\right)}^{2}}{\nu_{l}^{2}}\right).$$
  Equation (9), can be rewritten as:
  (10) $${\dot{f}}_{l}\left(t\right)=-\frac{2\left(t-m_{l}\right)}{\nu_{l}^{2}}f_{l}\left(t\right).$$
  Then from (8) and (10), $\dot{\hat{\chi }}\left(t,\theta ,m,\nu \right)$ is rewritten as:
  (11) $$\dot{\hat{\chi }}\left(t,\theta ,m,\nu \right)=\sum_{l=1}^{M}\theta_{l}\frac{-\frac{2\left(t-m_{l}\right)}{\nu_{l}^{2}}+f_{l}\left(t\right)\sum_{l=1}^{M}\frac{2\left(t-m_{l}\right)}{\nu_{l}^{2}}}{\sum_{l=1}^{M}f_{l}\left(t\right)}.$$
  Similarly, from (11), $\ddot{\hat{\chi }}\left(t,\theta ,m,\nu \right)$ is obtained as:
  (12) $$\begin{array}{c} \ddot{\hat{\chi }}\left(t,\theta ,m,\nu \right)=\\ \sum_{l=1}^{M}\theta_{l}\left(\begin{array}{c} \left[-\frac{2t}{\nu_{l}^{2}}+{\dot{f}}_{l}\left(t\right)\sum_{l=1}^{M}\frac{2\left(t-m_{l}\right)}{\nu_{l}^{2}}+f_{l}\left(t\right)\sum_{l=1}^{M}\frac{2t}{\nu_{l}^{2}}\right]\sum_{l=1}^{M}f_{l}\left(t\right)\\ -\left[\frac{2\left(t-m_{l}\right)}{\nu_{l}^{2}}+f_{l}\left(t\right)\sum_{l=1}^{M}\frac{2\left(t-m_{l}\right)}{\nu_{l}^{2}}\right]\sum_{l=1}^{M}{\dot{f}}_{l}\left(t\right) \end{array}\right)/{\left[\sum_{l=1}^{M}f_{l}\left(t\right)\right]}^{2}. \end{array}$$
  Considering (10), the Equation (12) is rewritten as:
  (13) $$\begin{array}{c} \ddot{\hat{\chi }}\left(t,\theta ,m,\nu \right)=\\ \sum_{l=1}^{M}\theta_{l}\left(\begin{array}{c} \left[-\frac{2t}{\nu_{l}^{2}}+{\dot{f}}_{l}\left(t\right)\sum_{l=1}^{M}\frac{2\left(t-m_{l}\right)}{\nu_{l}^{2}}+f_{l}\left(t\right)\sum_{l=1}^{M}\frac{2t}{\nu_{l}^{2}}\right]-\\ \left[\frac{2\left(t-m_{l}\right)}{\nu_{l}^{2}}+f_{l}\left(t\right)\sum_{l=1}^{M}\frac{2\left(t-m_{l}\right)}{\nu_{l}^{2}}\right]\sum_{l=1}^{M}\frac{2\left(t-m_{l}\right)}{\nu_{l}^{2}} \end{array}\right)/\sum_{l=1}^{M}f_{l}\left(t\right). \end{array}$$

## 4. Learning Method

The suggested T2-FLS is optimized through the SCKF. To apply SCKF on learning of T2-FLS such that the cost function (3) to be minimized, the following state-space representation is taken to account:

(14) $$\begin{array}{c} \xi_{k+1}=\xi_{k}+N_{k}\\ J_{k+1}=J_{k}\left({\hat{\chi }}_{k}|\xi_{k}\right)+W_{k}, \end{array}$$

where $\lambda \left(t\right)$ and $W\left(t\right)$ are the Gaussian noise with covariance *R* and *Q* and zeros mean and ξ is the vector of parameters of T2-FLS that includes rule parameters:

(15) $$\xi ={\left[\theta_{1}^{},\ldots ,\theta_{M}^{}\right]}^{T}.$$

The learning algorithm is presented as follows.

(1) Consider error covariance as $\phi_{k-1}$ at sample time k−1 and compute cubature points $C_{h}$, h=1,…,6M as:
  (16) $$C_{h,k-1}=\phi_{k-1}\varsigma_{h}+\xi_{k-1},$$
  where, *M* is the number of rules and $\varsigma_{h}$ is:
  (17) $$\varsigma_{h}=\left\{\begin{array}{c} \begin{array}{c} \sqrt{3M}{\left[\begin{array}{ccccc} 0 & \cdots & \overset{h-th\;\;element}{1} & \cdots & 0 \end{array}\right]}^{T}\\ h=1,2,\ldots ,3M \end{array}\\ \begin{array}{c} \sqrt{3M}{\left[\begin{array}{ccccc} 0 & \cdots & \overset{h-th\;\;element}{-1} & \cdots & 0 \end{array}\right]}^{T}\\ h=3M+1,\ldots ,6M. \end{array} \end{array}\right.$$
(2) For each $w_{\iota }$ in (16), evaluate the cost function *J* as:
  (18) $$\begin{array}{c} J_{h}=\frac{1}{N}\sum_{i=1}^{N}{\left({\ddot{\hat{\chi }}}_{i}\left(t|C_{h}\right)+\sum_{k=1}^{n}{\dot{\hat{\chi }}}_{i}\left(\gamma_{k}t|C_{h}\right)/P_{k,i}\left(t\right)+{\hat{\chi }}_{i}\left(t|C_{h}\right)/G_{i}-F\left(t\right)\right)}^{2}\\ \;\;\;\;\;\;\;\;\;\;\;\;\;\;\;\;\;\;+\frac{1}{2}\left({\hat{\chi }}_{0}^{2}\left(t|C_{h}\right)+{\dot{\hat{\chi }}}_{0}^{2}\left(t|C_{h}\right)\right), \end{array}$$
  where, h=1,…,6M.
(3) From (18), estimate $J_{m}$ as the mean of $J_{h},\;h=1,\ldots ,6M$:
  (19) $$J_{m}=\sum_{h=1}^{6M}J_{h}/6M.$$
(4) Define $Z_{k-1}$ as:
  (20) $$\begin{array}{c} Z_{k-1}=\frac{1}{\sqrt{6M}}\left[J_{1,k-1}-J_{m,k-1},\;\;\;J_{2,k-1}-J_{m,k-1},\;\;\right.\\ \;\;\;\;\;\;\;\;\;\;\;\;\;\;\;\;\;\;\;\;\;\;\;\;\;\;\;{\left.\cdots ,\;\;\;\;J_{6M,k-1}-J_{m,k-1}\right]}^{T}. \end{array}$$
(5) From (20), compute the square-root of covariance matrix as:
  (21) $$\phi_{zz,k-1}=\mathrm{Tria}\left(\left[\begin{array}{cc} Z_{k-1} & S_{R,k} \end{array}\right]\right),$$
  where $\mathrm{Tria}\left(\;\cdot \;\right)$ represents triangularization and $S_{R,k-1}$ is the square root of $R_{k-1}$.
(6) Compute cross-covariance $\pi_{\xi z,k-1}$ as:
  (22) $$\pi_{\xi z,k-1}=\zeta_{k-1}Z_{k-1}^{T},$$
  where
  (23) $$\begin{array}{c} \zeta_{k-1}=\frac{1}{\sqrt{6M}}\left[C_{1,k-1}-\xi_{k-1},C_{2,k-1}-\xi_{k-1},\;\right.\\ \;\;\;\;\;\;\;\;\;\left.\cdots ,\;\;\;\;\;\;\;\;\;C_{6M,k-1}-\xi_{k-1}\right]. \end{array}$$
(7) Obtain Kalman gain as:
  (24) $$\kappa_{k}=\left(\pi_{\xi z,k-1}/\phi_{zz,k-1}^{T}\right)/\phi_{zz,k-1}^{T}.$$
(8) Update ξ as:
  (25) $$\xi_{k}=\xi_{k-1}-\kappa_{k}J_{m}.$$
(9) Update error covariance as:
  (26) $$S_{R,k}=\mathrm{Tria}\left(\left[\begin{array}{cc} \zeta_{k-1}-\kappa_{k}Z_{k-1} & \kappa_{k}S_{R,k-1} \end{array}\right]\right).$$

## 5. Stability and Convergence Analysis

To prove the stability and convergence of the suggested algorithm, the Lyapunov approach [40,41] is used. To apply Lyapunov approach, the following Lyapunov function is defined:

(27) $$V\left(t\right)=\frac{1}{N}\sum_{i=1}^{N}{\left({\ddot{\hat{\chi }}}_{i}\left(t\right)+\sum_{k=1}^{n}{\hat{\chi }}_{i}\left(\gamma_{k}t\right)/P_{k,i}\left(t\right)+{\hat{\chi }}_{i}\left(t\right)/G_{i}-F\left(t\right)\right)}^{2}.$$

Time difference of *V*, results in:

(28) $$\begin{array}{c} V\left(t\right)-V\left(t-1\right)=\frac{1}{N}\sum_{i=1}^{N}{\left({\ddot{\hat{\chi }}}_{i}\left(t\right)+\sum_{k=1}^{n}{\hat{\chi }}_{i}\left(\gamma_{k}t\right)/P_{k,i}\left(t\right)+{\hat{\chi }}_{i}\left(t\right)/G_{i}\left(t\right)-F\left(t\right)\right)}^{2}\\ \;\;\;-\frac{1}{N}\sum_{i=1}^{N}{\left({\ddot{\hat{\chi }}}_{i}\left(t-1\right)+\sum_{k=1}^{n}{\hat{\chi }}_{i}\left(\gamma_{k}\left(t-1\right)\right)/P_{k,i}\left(t-1\right)+{\hat{\chi }}_{i}\left(t-1\right)/G_{i}\left(t-1\right)-F\left(t-1\right)\right)}^{2}. \end{array}$$

Considering small sample time, eq.dv1 can be simplified as:

(29) $$\begin{array}{c} V\left(t\right)-V\left(t-1\right)\leq \\ \frac{1}{N}\sum_{i=1}^{N}\left(\left|{\ddot{\hat{\chi }}}_{i}\left(t\right)\right|-\left|{\ddot{\hat{\chi }}}_{i}\left(t-1\right)\right|+\sum_{k=1}^{n}\left(\begin{array}{c} \left[\left|{\hat{\chi }}_{i}\left(\gamma_{k}t\right)\right|-\left|{\hat{\chi }}_{i}\left(\gamma_{k}\left(t-1\right)\right)\right|\right]/P_{k,i}\left(t\right)+\\ \left[\left|{\hat{\chi }}_{i}\left(t\right)\right|-\left|{\hat{\chi }}_{i}\left(t-1\right)\right|\right]/G_{i}\left(t\right) \end{array}\right)\right). \end{array}$$

From (13), ${\ddot{\hat{\chi }}}_{i}\left(t\right)-{\ddot{\hat{\chi }}}_{i}\left(t-1\right)$ is obtained as:

(30) $$\begin{array}{c} {\ddot{\hat{\chi }}}_{i}\left(t\right)-{\ddot{\hat{\chi }}}_{i}\left(t-1\right)=\\ \begin{array}{c} \sum_{l=1}^{M}\left[\theta_{l}\left(t\right)-\theta_{l}\left(t-1\right)\right]\;\cdot \;\\ \left(\begin{array}{c} \left[-\frac{2t}{\nu_{l}^{2}}+{\dot{f}}_{l}\left(t\right)\sum_{l=1}^{M}\frac{2\left(t-m_{l}\right)}{\nu_{l}^{2}}+f_{l}\left(t\right)\sum_{l=1}^{M}\frac{2t}{\nu_{l}^{2}}\right]-\\ \left[\frac{2\left(t-m_{l}\right)}{\nu_{l}^{2}}+f_{l}\left(t\right)\sum_{l=1}^{M}\frac{2\left(t-m_{l}\right)}{\nu_{l}^{2}}\right]\sum_{l=1}^{M}\frac{2\left(t-m_{l}\right)}{\nu_{l}^{2}} \end{array}\right)/\sum_{l=1}^{M}f_{l}\left(t\right). \end{array} \end{array}$$

From (11), ${\dot{\hat{\chi }}}_{i}\left(\gamma_{k}t\right)-{\hat{\chi }}_{i}\left(\gamma_{k}\left(t-1\right)\right)$ is written as:

(31) $$\begin{array}{c} {\dot{\hat{\chi }}}_{i}\left(\gamma_{k}t\right)-{\hat{\chi }}_{i}\left(\gamma_{k}\left(t-1\right)\right)\\ =\sum_{l=1}^{M}\left[\theta_{l}\left(t\right)-\theta_{l}\left(t-1\right)\right]\frac{-\frac{2\left(t-m_{l}\right)}{\nu_{l}^{2}}+f_{l}\left(t\right)\sum_{l=1}^{M}\frac{2\left(t-m_{l}\right)}{\nu_{l}^{2}}}{\sum_{l=1}^{M}f_{l}\left(t\right)}. \end{array}$$

From (7), ${\hat{\chi }}_{i}\left(t\right)-{\hat{\chi }}_{i}\left(t-1\right)$ is computed as:

(32) $${\hat{\chi }}_{i}\left(t\right)-{\hat{\chi }}_{i}\left(t-1\right)=\sum_{l=1}^{M}\left[\theta_{l}\left(t\right)-\theta_{l}\left(t-1\right)\right]\psi_{l}\left(t,m,\nu \right).$$

From (29) and Equations (31) and (32), one has:

(33) $$\begin{array}{c} V\left(t\right)-V\left(t-1\right)=\frac{1}{N}\sum_{i=1}^{N}\sum_{l=1}^{M}\left[\theta_{l}\left(t\right)-\theta_{l}\left(t-1\right)\right]\;\cdot \;\\ \begin{array}{c} \left\{\left|\left(\begin{array}{c} \left[-\frac{2t}{\nu_{l}^{2}}+{\dot{f}}_{l}\left(t\right)\sum_{l=1}^{M}\frac{2\left(t-m_{l}\right)}{\nu_{l}^{2}}+f_{l}\left(t\right)\sum_{l=1}^{M}\frac{2t}{\nu_{l}^{2}}\right]-\\ \left[\frac{2\left(t-m_{l}\right)}{\nu_{l}^{2}}+f_{l}\left(t\right)\sum_{l=1}^{M}\frac{2\left(t-m_{l}\right)}{\nu_{l}^{2}}\right]\sum_{l=1}^{M}\frac{2\left(t-m_{l}\right)}{\nu_{l}^{2}} \end{array}\right)/\sum_{l=1}^{M}f_{l}\left(t\right)\right|+\right.\\ \left.\;\;\;\;\;\;\;\;\;\;\;\;\;\sum_{k=1}^{n}\left(\begin{array}{c} \left|\frac{-\frac{2\left(t-m_{l}\right)}{\nu_{l}^{2}}+f_{l}\left(t\right)\sum_{l=1}^{M}\frac{2\left(t-m_{l}\right)}{\nu_{l}^{2}}}{\sum_{l=1}^{M}f_{l}\left(t\right)}\right|/P_{k,i}\left(t\right)+\\ \left|\psi_{l}\left(t,m,\nu \right)\right|/G_{i}\left(t\right) \end{array}\right)\right\}. \end{array} \end{array}$$

From (25) and (33), one has:

(34) $$\begin{array}{c} V\left(t\right)-V\left(t-1\right)\leq \frac{1}{N}\sum_{i=1}^{N}\sum_{l=1}^{M}-\kappa J_{m}\;\cdot \;\\ \begin{array}{c} \left\{\left|\left(\begin{array}{c} \left[-\frac{2t}{\nu_{l}^{2}}+{\dot{f}}_{l}\left(t\right)\sum_{l=1}^{M}\frac{2\left(t-m_{l}\right)}{\nu_{l}^{2}}+f_{l}\left(t\right)\sum_{l=1}^{M}\frac{2t}{\nu_{l}^{2}}\right]-\\ \left[\frac{2\left(t-m_{l}\right)}{\nu_{l}^{2}}+f_{l}\left(t\right)\sum_{l=1}^{M}\frac{2\left(t-m_{l}\right)}{\nu_{l}^{2}}\right]\sum_{l=1}^{M}\frac{2\left(t-m_{l}\right)}{\nu_{l}^{2}} \end{array}\right)/\sum_{l=1}^{M}f_{l}\left(t\right)\right|+\right.\\ \left.\;\;\;\;\;\;\;\;\;\;\;\;\;\sum_{k=1}^{n}\left(\begin{array}{c} \left|\frac{-\frac{2\left(t-m_{l}\right)}{\nu_{l}^{2}}+f_{l}\left(t\right)\sum_{l=1}^{M}\frac{2\left(t-m_{l}\right)}{\nu_{l}^{2}}}{\sum_{l=1}^{M}f_{l}\left(t\right)}\right|/P_{k,i}\left(t\right)+\\ \left|\psi_{l}\left(t,m,\nu \right)\right|/G_{i}\left(t\right) \end{array}\right)\right\}. \end{array} \end{array}$$

From the fact that $J_{m}>0$, it is concluded that $V\left(t\right)-V\left(t-1\right)\leq 0$ and from the Lyapunov theorem, the stability and boundedness of the cost function is derived.

## 6. Evaluation Index

To evaluate the accuracy and robustness of the suggested algorithm, the following indexes are defined.

(35) $$\mathrm{RMSE}=\sqrt{\frac{1}{N}\sum_{i=1}^{N}{\left(\chi_{i}-{\hat{\chi }}_{i}\right)}^{2}},$$

(36) $$\mathrm{TIC}=\sqrt{\frac{1}{N}\sum_{i=1}^{N}{\left(\chi_{i}-{\hat{\chi }}_{i}\right)}^{2}}/\left(\sqrt{\frac{1}{N}\sum_{i=1}^{N}\chi_{i}^{2}}+\sqrt{\frac{1}{N}\sum_{i=1}^{N}{\hat{\chi }}_{i}^{2}}\right),$$

(37) $$\mathrm{VAR}=\sum_{i=1}^{N}{\left(\chi -{\hat{\chi }}_{i}\right)}^{2}/\left(N-1\right),$$

where *N* is the number of sample times, $\chi_{i}$ and ${\hat{\chi }}_{i}$ are the exact and estimated solutions and RMSE, TIC and VAR are root mean square error, inequality coefficient of Theil index, and variance, respectively.

## 7. Simulations

By several statistical analyses, the accuracy of the suggested algorithm is examined.

**Example** **1.** *For the first examination, an SMDE is considered as:*

(38) $$\ddot{\chi }\left(t\right)+\frac{1}{t}\dot{\chi }\left(t/2\right)+\frac{1}{t^{2}}\dot{\chi }\left(t/4\right)+\frac{1}{1-t}\chi \left(t\right)=F\left(t\right),\;\;0<t\leq 1,$$

*where*

(39) $$F\left(t\right)=\frac{1}{t}\alpha \cos\left(\alpha t/2\right)+\frac{1}{t^{2}}\alpha \cos\left(\alpha t/4\right)+\frac{\alpha^{2}t+\left(1-\alpha^{2}\right)}{1-t}\sin\left(\alpha t\right),$$

*where, α=π. The real solution of (38), is $\sin\left(\alpha t\right)$. To estimate the solution by the suggested method, the cost function is given as:*

(40) $$\begin{array}{c} J=\\ \frac{1}{N}\sum_{u=0}^{N}{\left(\left(1-u\right)u^{2}\ddot{\hat{\chi }}\left(u|\xi \right)+\left(1-u\right)u\dot{\hat{\chi }}\left(\frac{u}{2}|\xi \right)+\left(1-u\right)\dot{\hat{\chi }}\left(\frac{u}{4}|\xi \right)-\left(1-u\right)u^{2}F\left(u\right)\right)}^{2}\\ N=1/\tau ,\;\;\;\;\;\tau =0.05. \end{array}$$

**Figure 3:**
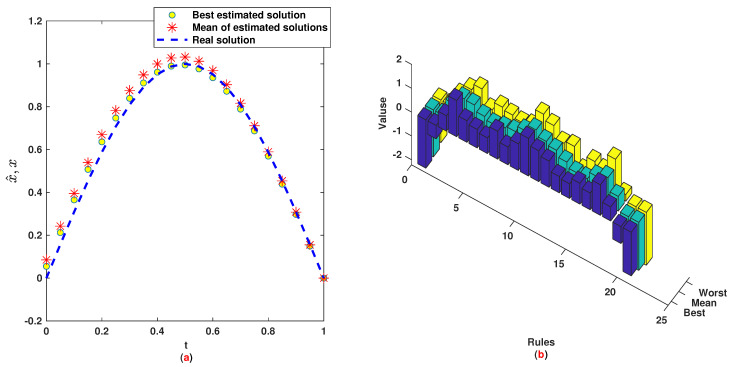
Example 1: (**a**): Solution performance; (**b**): Weights of NN.

**Figure 4:**
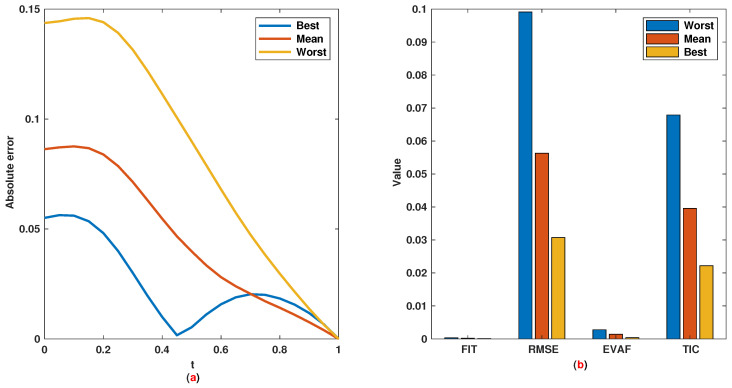
Example 1: (**a**): Absolute error; (**b**): The values of FIT, RMSE, VAR and TIC.

**Figure 5:**
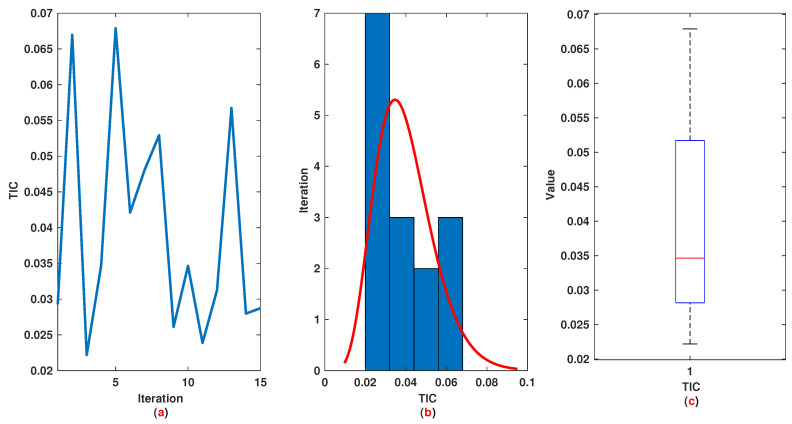
Example 1: (**a**): The value of TIC at each iteration; (**b**): Histogram plot for TIC; (**c**): Box plot for TIC.

**Figure 6:**
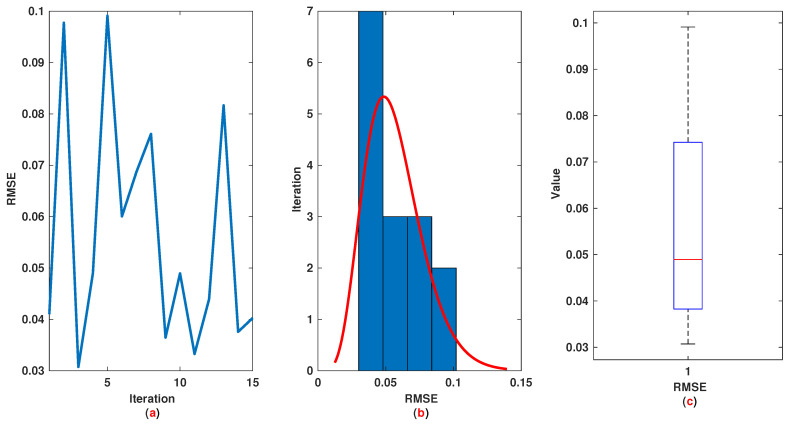
Example 1: (**a**): The value of RMSE at each iteration; (**b**): Histogram plot for RMSE; (**c**): Box plot for RMSE.

**Figure 7:**
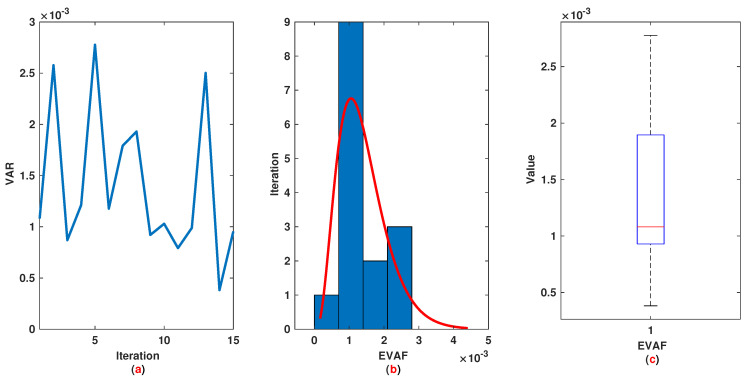
Example 1: (**a**): The value of VAR at each iteration; (**b**): Histogram plot for VAR; (**c**): Box plot for VAR.

**Figure 8:**
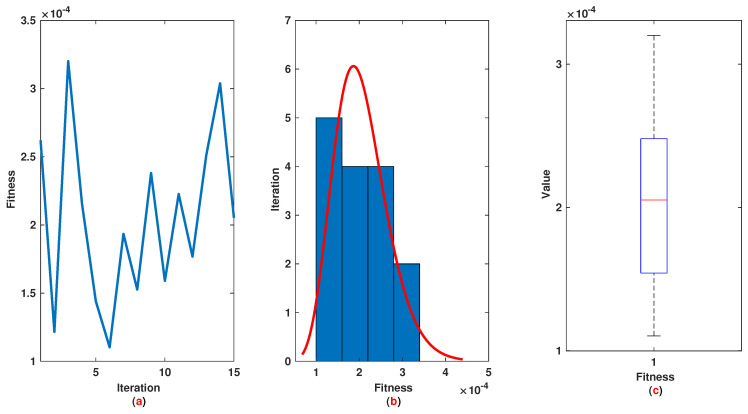
Example 1: (**a**): The value of FIT at each iteration; (**b**): Histogram plot for FIT; (**c**): Box plot for FIT.

*The time range $\left[0,1\right]$ is divided into 21 sections and then we have 21 rules. The standard division of each MF is considered to be 0.1. The trajectories of the output of T2-FLS (approximated solution), mean of approximated solutions and exact solution are depicted in Figure 3a and the corresponding rule parameters are shown in Figure 3b. The absolute error is depicted in Figure 4a and the values of FIT, RMSE, VAR and TIC are shown in Figure 4b. The statistical analyses for TIC, RMSE, VAR and FIT are given in Figure 5, Figure 6, Figure 7 and Figure 8. One can observe that the metrics of TIC, RMSE, VAR and FIT are in the favorable level and the trajectory of the approximated solution well tracks the exact solution x(t)=sin(αt).*

For the accuracy of the suggested approach to be well seen, the values of interquartile range (IR), median (Med), minimum (Min) and mean of absolute error at each sample time are provided in Table 1. One can see that the values of mean and IR items are in range of $10^{-3}$ to $10^{-2}$ that indicate an accurate and robust solution.

**Example** **2.** *In this Example, the following SMDE is considered:*

(41) $$\ddot{\chi }\left(t\right)+\frac{1}{t}\dot{\chi }\left(t/2\right)+\frac{1}{t^{2}}\dot{\chi }\left(t/4\right)+\frac{1}{1-t}\chi \left(t\right)=F\left(t\right),\;\;0<t\leq 1,$$

*where*

(42) $$F\left(t\right)=\frac{1}{t}\exp\left(t/2\right)+\frac{1}{t^{2}}\exp\left(t/4\right)+\frac{2-t}{1-t}\exp\left(t\right).$$

*The cost function is:*

(43) $$\begin{array}{c} J=\\ \frac{1}{N}\sum_{u=0}^{N}{\left(\left(1-u\right)u^{2}\ddot{\hat{\chi }}\left(u|w\right)+\left(1-u\right)u\dot{\hat{\chi }}\left(\frac{u}{2}|w\right)+\left(1-u\right)\dot{\hat{\chi }}\left(\frac{u}{4}|w\right)-\left(1-u\right)u^{2}F\left(u\right)\right)}^{2}\\ \;\;\;\;\;\;\;\;\;\;\;+\frac{1}{2}\left({\left(\hat{\chi }\left(0|w\right)-1\right)}^{2}+{\left(\dot{\hat{\chi }}\left(0|w\right)\right)}^{2}\right)\\ N=1/\tau ,\tau =0.05. \end{array}$$

**Figure 9:**
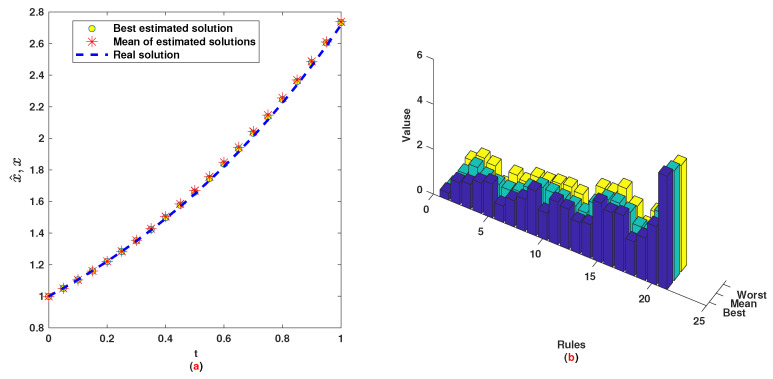
Example 2: (**a**): Solution performance; (**b**): Weights of NN.

**Figure 10:**
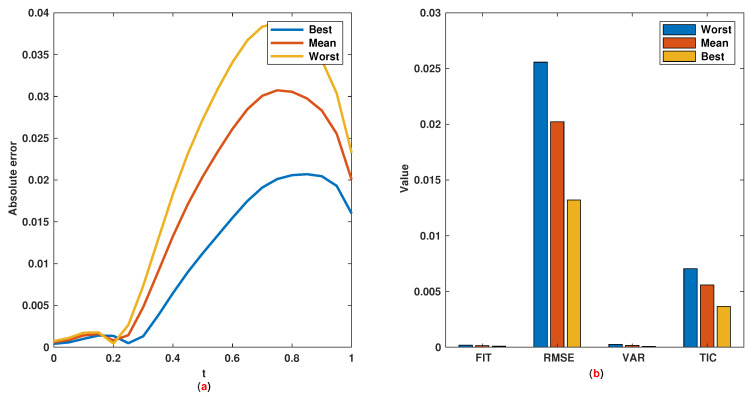
Example 2: (**a**): Absolute error; (**b**): The values of FIT, RMSE, VAR and TIC.

**Figure 11:**
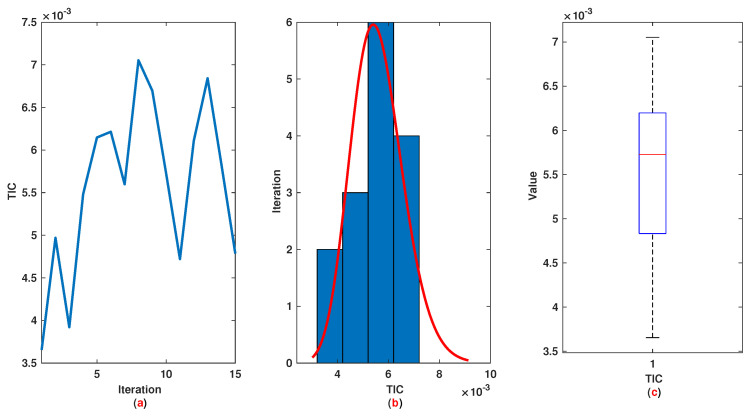
Example 2: (**a**): The value of TIC at each iteration; (**b**): Histogram plot for TIC; (**c**): Box plot for TIC.

**Figure 12:**
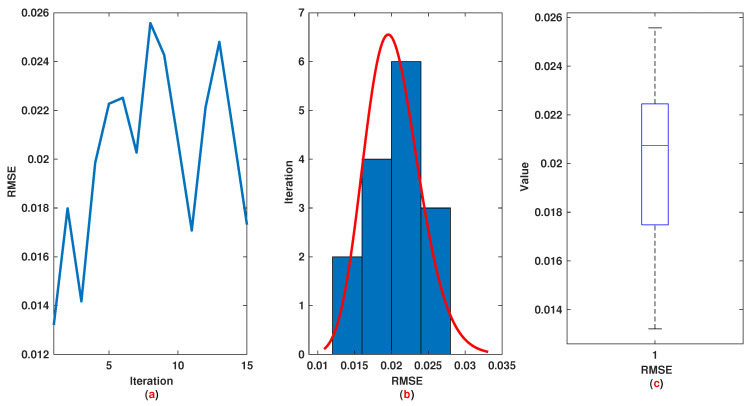
Example 2: (**a**): The value of RMSE at each iteration; (**b**): Histogram plot for RMSE; (**c**): Box plot for RMSE.

**Figure 13:**
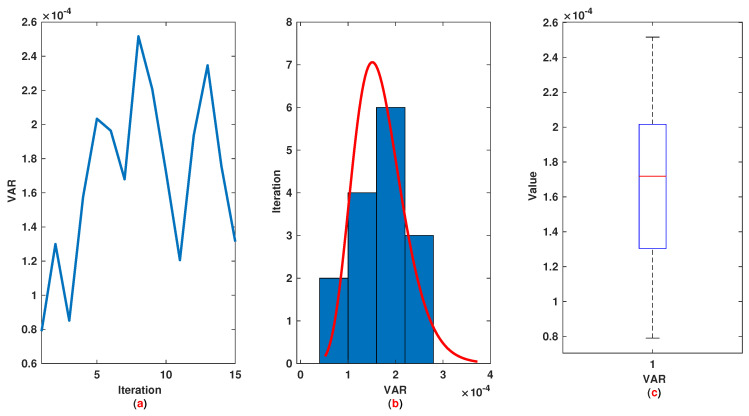
Example 2: (**a**): The value of VAR at each iteration; (**b**): Histogram plot for VAR; (**c**): Box plot for VAR.

**Figure 14:**
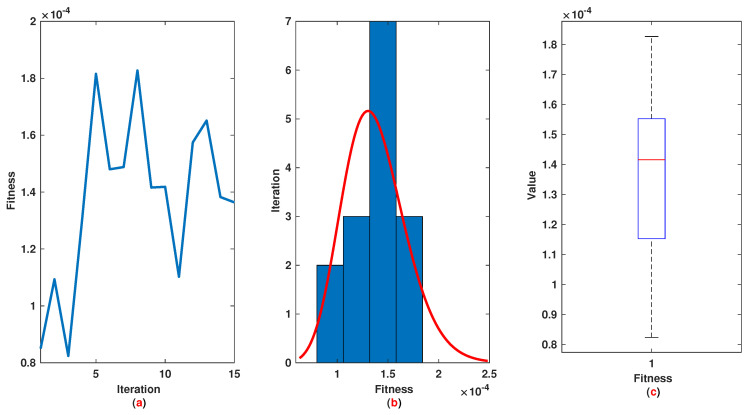
Example 2: (**a**): The value of FIT at each iteration; (**b**): Histogram plot for FIT; (**c**): Box plot for FIT.

*Similar to Example 1, we have 21 rules. The trajectories of the output of T2-FLS (approximated solution), the mean of approximated solutions and exact solution are depicted in Figure 9a and the corresponding rule parameters are shown in Figure 9b. The absolute error is depicted in Figure 10a and the values of FIT, RMSE, VAR and TIC are shown in Figure 10b. The statistical analysis for TIC, RMSE, VAR and FIT are given in Figure 11, Figure 12, Figure 13 and Figure 14. One can observe that the metrics of TIC, RMSE, VAR and FIT are in the favorable level and the trajectory of the approximated solution well tracks the exact solution x(t)=exp(t).*

Similar to Example 1, in order for the accuracy of the suggested approach to be well seen, the values of interquartile range (IR), median (Med), minimum (Min) and mean of absolute error at each sample time are provided in Table 2. One can see that the values of mean and IR items are in the range of $10^{-3}$ to $10^{-2}$ that indicate an accurate and robust solution.

**Remark** **1.** *The main hyperparameters of the suggested algorithm are the number of rules and initial covariance matrices. For stability considerations, the initial covariance matrices are chosen to be relatively small. The number of rules is equal to the number of membership functions for input t. To determine the number of rules, the time range $t\in \left[0,T\right]$ is divided into M sections and for each section one Gaussian membership function with a certain center and an uncertain standard division is considered. The number of rules can affect the accuracy but it depends on the size of sample time. For a smaller sample time, more rules can be taken into account.*

**Example** **3.** *In this section, the performance of the suggested algorithm is compared with other similar techniques in the literature [29]. In Reference [29], a simple NN is learned by genetic algorithm to find a solution for a pantograph system. The following MDE is taken into account [29]:*

(44) $$\dot{\chi }=\frac{1}{2}e^{t/2}\chi \left(t/2\right)+\frac{1}{2}\chi \left(t\right),\;\chi \left(0\right)=1.$$

*The exact solution of (44) is $\chi \left(t\right)=e^{t}$. The number of rules in T2-FLS is considered to be 10, the same as the number of neurons in Reference [29]. The numerical comparison, with the method of Reference [29], is given in Table 3. Although the results are close to each other in this Example, the main advantage of the suggested method is that the results of the suggested method are obtained in only one epoch. However, in the genetic algorithm presented in Reference [29], the learning process of NN is repeated several times until a reasonable result is achieved. The evolutionary based learning techniques are not suitable for online applications because of the high level of computational cost and lack of stability guarantee.*

## 8. Conclusions

In this paper, a new approach on the basis of fuzzy neural networks and SCKF is introduced for finding a numerical solution for multi-pantograph singular differential equations. The proposed learning method is stable and this property is shown by a new approach on the basis of the Lypunov theorem. Two simulations are provided to demonstrate the efficiency of the designed solver. Several statistical analyses are given to verify the effectiveness of the introduced algorithm such as the analysis of RMSE, Interquartile Range, Theil’s Inequality Index and Variance metrics. The metrics of TIC, RMSE, VAR and FIT are shown in the favorable level and the trajectory approximated solution well tracks the exact solution. Also, the performance of the suggested method is compared with the other similar techniques in the literature. It is shown that the proposed technique results in better accuracy despite less computational cost in contrast to the evolutionary based learning genetic algorithm.

## Figures and Tables

**Table 1:** Example 1: Statistical analysis.

*t*	Min	Mean	Med	IR
0	0.0218	0.0776	0.0823	0.0451
0.0500	0.0228	0.0786	0.0830	0.0448
0.1000	0.0223	0.0788	0.0835	0.0455
0.1500	0.0200	0.0774	0.0827	0.0473
0.2000	0.0154	0.0736	0.0799	0.0498
0.2500	0.0089	0.0672	0.0746	0.0519
0.3000	0.0014	0.0589	0.0675	0.0523
0.3500	0.0040	0.0505	0.0595	0.0520
0.4000	0.0001	0.0430	0.0514	0.0500
0.4500	0.0030	0.0373	0.0434	0.0416
0.5000	0.0039	0.0323	0.0354	0.0328
0.5500	0.0003	0.0279	0.0278	0.0202
0.6000	0.0006	0.0242	0.0231	0.0139
0.6500	0.0042	0.0211	0.0209	0.0129
0.7000	0.0026	0.0179	0.0167	0.0159
0.7500	0.0001	0.0149	0.0124	0.0138
0.8000	0.0019	0.0121	0.0120	0.0127
0.8500	0.0012	0.0092	0.0091	0.0112
0.9000	0.0000	0.0062	0.0057	0.0087
0.9500	0.0003	0.0033	0.0030	0.0053
1.0000	0.0000	0.0000	0.0000	0.0000

**Table 2:** Example 2: Statistical analysis.

*t*	Min	Mean	Med	IR
0	0.0004	0.0006	0.0006	0.0001
0.0500	0.0006	0.0009	0.0009	0.0002
0.1000	0.0010	0.0014	0.0015	0.0003
0.1500	0.0013	0.0016	0.0016	0.0002
0.2000	0.0000	0.0008	0.0008	0.0005
0.2500	0.0002	0.0014	0.0013	0.0014
0.3000	0.0013	0.0048	0.0047	0.0025
0.3500	0.0038	0.0090	0.0092	0.0038
0.4000	0.0065	0.0133	0.0137	0.0050
0.4500	0.0090	0.0171	0.0176	0.0059
0.5000	0.0112	0.0204	0.0210	0.0067
0.5500	0.0134	0.0234	0.0240	0.0073
0.6000	0.0155	0.0261	0.0268	0.0077
0.6500	0.0175	0.0285	0.0292	0.0079
0.7000	0.0191	0.0301	0.0309	0.0079
0.7500	0.0201	0.0307	0.0316	0.0075
0.8000	0.0206	0.0306	0.0314	0.0069
0.8500	0.0207	0.0298	0.0306	0.0064
0.9000	0.0205	0.0283	0.0291	0.0057
0.9500	0.0193	0.0255	0.0262	0.0041
1.0000	0.0157	0.0201	0.0205	0.0028

**Table 3:** Example 3: Comparison.

*t*	Exact Solution	Proposed Method	Method of Reference [29]
0	1.0000	1.0000	1.0000
0.1	1.1052	1.1051	1.1051
0.2	1.2214	1.2213	1.2213
0.3	1.3499	1.3498	1.3497
0.4	1.4918	1.4917	1.4917
0.5	1.6487	1.6486	1.6486
0.6	1.8221	1.8221	1.8220
0.7	2.0138	2.0137	2.0136
0.8	2.2255	2.2254	2.2253
0.9	2.4596	2.4595	2.4594
1	2.7183	2.7181	2.7181

## Abbreviations

SCKF	Square root cubature Kalman filter
SMDE	Singular multi-pantograph differential equations
T2-FLS	Type-2 fuzzy logic system
FLS	Fuzzy logic system
NN	Neural network
MDE	Multi-pantograph differential equation
RMSE	Root mean square error
TIC	Inequality coefficient of Theil index
VAR	variance
FIT	Fitness
IR	Interquartile range
Med	Median
Min	Minimum
*M*	Number of rules
$m_{l}$	Center of *l*-th Gaussian membership function
${\bar{\nu }}_{l}$	Upper standard division
${\underline{\nu }}_{l}$	Lower standard division
*J*	Cost function
$\psi_{l}$	*l*-th rule firing
ϕ	Covariance matrix
κ	Kalman gain
*N*	Number of data samples
